# PKC alpha affects cell cycle progression and proliferation in human RPE cells through the downregulation of p27^kip1^

**Published:** 2009-12-10

**Authors:** Qianying Gao, Juan Tan, Ping Ma, Jian Ge, Yaqin Liu, Xuerong Sun, Lian Zhou

**Affiliations:** State Key Laboratory of Ophthalmology, Zhongshan Ophthalmic Center, Sun Yat-sen University, Guangzhou, China

## Abstract

**Purpose:**

Protein kinase C (PKC) plays an important role in the regulation of retinal pigment epithelium (RPE) cell proliferation. In this study, we investigated which of these isozymes could be responsible for the cell cycle and proliferation in human RPE cells.

**Methods:**

The effect of PKC activators on human RPE cell cycle progression was tested by flow cytometry. To identify the isoform of PKC responsible for the increased progression of the cells through the cell cycle, we monitored the effect of phorbol 12-myristate 13-acetate (PMA) on the subcellular localization of the nine PKC isoforms expressed in RPE cells. To evaluate the molecular mechanism by which PKC_α_ induces cell cycle progression, we examined the transcript, protein, and cellular levels of cell cycle regulatory proteins using RT–PCR, western blotting, and a confocal microscope, respectively.

**Results:**

We demonstrated that PKC activation by PMA affected cell cycle progression in RPE cells. Of the nine PKC isoforms that were present in RPE cells, we found PKC_α_ was both necessary and sufficient to promote cell cycle progression after being stimulated with PMA. Decreased PKC_α_ expression resulted in a significant decrease in cell proliferation. The only cell cycle-regulatory molecule whose expression was rapidly altered and decreased by PKC_α_ activity was the cyclin- dependent kinase (CDK) inhibitor p27^kip1^.

**Conclusions:**

These results suggest that PKC_α_ affects cell cycle progression and proliferation in human RPE cells through the downregulation of p27^kip1^.

## Introduction

Protein kinase C (PKC) is a multigene family of phospholipid-dependent serine-threonine kinases that mediates the phosphorylation of numerous protein substrates in signal transduction. It plays a central role in cellular processes such as proliferation, differentiation, mitosis, and inflammatory reactions [1,2]. Up to now, at least 12 isoforms of PKC have been cloned to date, all displaying different enzymatic properties, tissue expression, and intracellular localization [3,4]. PKCs are divided into three major groups according to the variability of their regulatory domains. The classic PKCs (cPKC: PKC_α_, PKC_βI_, PKC_βII_, and PKC_γ_) require calcium, phosphatidylserine, and diacylglycerol (DAG), or phorbol esters, for full activation. The novel PKCs (nPKC: PKC_δ_, PKC_ε_, PKC_η_, PKC_θ_, and probably PKC_μ_ [5]) do not require calcium or their activation. The third group are the atypical PKCs (aPKC: PKC_ζ_, PKC_λ_ and PKC_ι_), whose activation depends on phosphatidylserine, but not on DAG, nor on calcium or phorbol esters. The differences in function of specific PKC isoforms are mainly due to their subcellular localization, their activation or inhibition by different stimuli, and transcriptional regulation [6,7].

It has been well documented that the PKC family is involved in the processes of proliferation, migration, phagocytosis, and gel contraction in retinal pigment epithelium (RPE) cells [8-14], which have all been implicated in the pathogenesis of proliferative vitreoretinopathy (PVR). For example, Harris et al. reported that hypericin, a specific inhibitor of PKC, could have potential as a therapeutic drug for PVR and that its antiproliferative and apoptotic effects on RPE cells in vitro were in part mediated by PKC [9]. Another study showed that the PKC inhibitor calphostin C dramatically affected the growth rate of RPE cells [10]. We have found that hypericin has potential as a therapeutic drug for PVR, potentially through its inhibition of the Ca^2+^ influx pathway [15]. Rabbit models have shown that intravitreal injection of hypericin is also a safe and effective means of reducing experimental PVR [16,17]. However, since the distribution of PKC isoforms is both tissue-specific and cell type-specific [18], the PKC activity is the sum of the isoforms expressed in that tissue. Therefore, data regarding the precise pattern of isoform expression in RPE cells could be informative with regard to their physiologic regulation and potential role in PVR [19]. Our previous study characterized the expression pattern of all 12 PKC isoforms and showed that ten isoforms (PKC_α_, PKC_βI_, PKC_βII_, PKC_δ_, PKC_ε_, PKC_θ_, PKC_μ_, PKC_ζ_, PKC_λ_, and PKC_ι_) were present in cultured human RPE cells [20]. This identification provides the first step toward elucidating their roles in RPE cell proliferation. In this study, we further investigated which of these isozymes could be responsible for the cell cycle in human RPE cells. Our results demonstrate that PKC_α_ controls proliferation and regulates cell cycle progression in RPE cells through the downregulation of cyclin-dependent kinase (CDK) inhibitor p27^kip1^.

## Methods

### Reagents

Trizol reagent was obtained from Life Technologies (Gaithersburg, MD). The SuperScript™ first strand synthesis system was obtained from Invitrogen (Carlsbad, CA). The enhanced chemiluminescence (ECL) kit for western blotting was from Cell Signaling (Danvers, MA). Rabbit polyclonal antibodies against p27 and phorbol 12-myristate 13-acetate (PMA) were obtained from Santa Cruz Biotechnology (Santa Cruz, CA). Monoclonal PKC_α_, PKC_γ_, PKC_δ_, PKC_ε_, PKC_η_, PKC_θ_, PKC_ι_, and PKC_λ_ antibodies were purchased from BD Systems (Torrance, CA). Monoclonal PKC_βI_, PKC_βII_, PKC_ζ_, and PKC_μ_ antibodies were from Sigma (St. Louis, MO). Anti-β actin was purchased from Boster Biologic Technology, LTD (Wuhan, China). Thymeleatoxin was from Biovision (Mountain View, CA). Small-interference (si) RNA-PKC_α_ was obtained from Ruibo Biotech (Guangzhou, China). Lipofectamine 2000 was purchased from Invitrogen.

### Human RPE cell culture

Human RPE cells were isolated from five human donors, age 23 to 40 years, within 24 h after death, which were obtained from the Zhongshan Ophthalmic Center, as previously described [20]. This project was approved by the Ethics Committee of the Zhongshan Ophthalmic Center, and followed the tenets of the Declaration of Helsinki. Briefly, the anterior segment, vitreous and neurosensory retina were removed and an eye cup was made. The RPE cells were immersed in a trypsin (0.05%)-EDTA (0.02%) solution at 37 °C for 1 h. Culture medium with 20% FBS was added, and the RPE were isolated and collected with a pipette, using a dissecting microscope. Isolated cells were centrifuged, resuspended and seeded to Corning culture plates in Dulbecco’s modified Eagle medium (DMEM) containing 10% fetal bovine serum, penicillin G (100 μ/ml), streptomycin sulfate (100 mg/ml), and 2mM L-glutamate in. Experimentation was performed using 70%–80% confluent cells at cell passage 3 to 8.

### Flow cytometry

Confluent RPE cells were stimulated with 100 nM PMA, 100 nM thymeleatoxin and DMEM (as control group) and collected at each time point, then incubated in PBS (8.00 g/l sodium chloride, 0.20 g/l potassium chloride, 1.56 g/l Na_2_HPO_4_.H_2_O, 0.20 g/l KH_2_PO_4_) containing 50 µg/ml/10^6^ cells RNase A and 50 µg/ml/10^6^ cells propidium iodide (PI) for 30 min at 37 °C. The cell cycle analysis of treated cells at each time point were done on FACScan Flow Cytometer (Becton Dickinson). The red fluorescence (PI) from cells were excited at 488 nm using channel 2. The distribution of cells in different phases of the cell cycle was obtained by analyzing fluorescence intensities with Lysis II software (Becton Dickinson).

### Reverse transcription-polymerase chain reaction

Total RNA was extracted using Trizol reagent according to the manufacturer’s procedure. The integrity of the RNA was checked by 2% agarose gel electrophoresis. Approximately 5 µg RNA was reverse-transcribed following the protocol of the SuperScript™ first-strand synthesis system. cDNAs encoding the cell cycle regulator genes were amplified by PCR as follows: denaturation for 30 s, annealing for 30 s and elongation at 72 °C for 60 s. Primer sequences were designed using Primer 3, as shown in Table 1. Each PCR was done a minimum of three times with each set of primers. PCR products were analyzed by agarose (2%) gel electrophoresis and ethidium bromide staining.

**Table 1:** Primers and PCR conditions of cell cycle regulator genes

**Name**	**Sequence**	**Product size (bp)**	**Tm (°C)**
CDK1	F: TTTTCAGAGCTTTGGGCACT	195	55
	R: CCATTTTGCCAGAAATTCGT		
CDK2	F: CATTCCTCTTCCCCTCATCA	173	57
	R: CAGGGACTCCAAAAGCTCTG		
CDK3	F: TTTGCAGAGATGGTGACTCG	167	57
	R: AGTCCCTTCCTGGTCCACTT		
CDK4	F: GAAACTCTGAAGCCGACCAG	213	57
	R: AGGCAGAGATTCGCTTGTGT		
Cyclin A	F: TTATTGCTGGAGCTGCCTTT	224	55
	R: CTCTGGTGGGTTGAGGAGAG		
Cyclin A1	F: ACCCCAAGAGTGGAGTTGTG	198	55
	R: GGAAGGCATTTTCTGATCCA		
Cyclin B1	F: CGGGAAGTCACTGGAAACAT	177	55
	R: AAACATGGCAGTGACACCAA		
Cyclin B2	F: TTGCAGTCCATAAACCCACA	218	55
	R: GAAGCCAAGAGCAGAGCAGT		
Cyclin C	F: AGGCCCCACTCTTATGTCCT	231	59
	R: TGGTGAAACCCCGTCTCTAC		
Cyclin D1	F: AACTACCTGGACCGCTTCCT	204	57
	R: CCACTTGAGCTTGTTCACCA		
Cyclin D2	F: TGGGGAAGTTGAAGTGGAAC	175	57
	R: ATCATCGACGGTGGGTACAT		
Cyclin D3	F: TGGATGCTGGAGGTATGTGA	190	55
	R: TGCACAGTTTTTCGATGGTC		
Cyclin E1	F: CAGATTGCAGAGCTGTTGGA	225	57
	R: TCCCCGTCTCCCTTATAACC		
Cyclin E2	F: CAGGTTTGGAGTGGGACAGT	199	59
	R: CTCCATTGCACACTGGTGAC		
P16	F: CTCTGGAGGACGAAGTTTGC	158	57
	R: CATTCCTCTTCCTTGGTTTCC		
P18	F: TGCACAAAATGGATTTGGAA	223	51
	R: GGGCAGGTTCCCTTCATTAT		
P19	F: CTGCAGGTCATGATGTTTGG	229	57
	R: CAGCAGTGTGACCCTCTTGA		
P27	F: ATGTCAAACGTGCGAGTGTC	152	57
	R: TCTCTGCAGTGCTTCTCCAA		
P21	F: GACACCACTGGAGGGTGACT	172	59
	R: CAGGTCCACATGGTCTTCCT		
P107	F: CCAGTGGTGTGGTCAATCAG	164	59
	R: GAACAGCGAGTTTGAGGAG		
Rb	F: GGAAGCAACCCTCCTAAACC	153	57
	R: TTTCTGCTTTTGCATTCGTG		
GAPDH	F: ACCCAGAAGACTGTGGATGG	415	55
	R: TGCTGTAGCCAAATTCGTTG		

RNA (5 µg) was reverse-transcribed following the protocol of the SuperScript™ first-strand synthesis system. cDNAs encoding the cell cycle regulator genes were amplified by PCR as follows: denaturation for 30 s, annealing for 30 s and elongation at 72 °C for 60 s. Primer sequences were designed using Primer 3.

### Preparation of cell extracts

The medium was removed and washed twice with ice-cold PBS. The human RPE cells were lysed with sample buffer that contained 60 mM Tris, pH 6.8, 2% (w/v) SDS, 100 mM 2-mercaptoethanol, and 0.01% (w/v) bromophenol blue [21]. The lysates were then incubated on ice for 30 min. The extracts were harvested using a cell scraper, then boiled for 5 min and stored at –20 °C.

### Western blot analysis

Cellular extracts from confluent human RPE cells were processed for western blot analysis [22]. Briefly, 40 µg of protein per well was loaded on a 12% sodium dodecyl sulfate- PAGE (SDS–PAGE) gel. Protein was electrotransferred to polyvinylidene difluoride membranes (Millipore) for 2 h at 350 mA, then blocked with a solution of Tris-buffered saline (TBS) containing 5% nonfat milk and 0.1% Tween-20 (TBST) for 1 h, and incubated with primary antibodies overnight at 4 °C. After three washes with TBST for 10 min at room temperature, the membranes were incubated with horseradish peroxidase-conjugated secondary antibody for 3 h at room temperature, and then washed one time with TBST for 30 min at room temperature. Localization of antibodies was detected by chemiluminescence using an ECL kit following the manufacturer’s instructions. Each PKC isoform was examined in a minimum of four independent experiments. As recommended by the supplier of the primary antibodies, we used mouse brain lysate as a positive control.

### Subcellular fractionation

Confluent cells were partitioned into soluble and particulate fractions, using a method previously described [23,24]. Briefly, cells were lysed in digitonin lysis buffer (as described in the previous section, but without Triton X-100) and homogenized for 10 s at 3300× g Digitonin-soluble (cytosolic) and insoluble (particulate) fractions were separated by ultracentrifugation at 100,000× g for 45 min at 4 °C. Supernatant was collected, and it formed the cytosolic fraction. The pellet was resuspended in digitonin buffer containing 1% Triton X-100, incubated on ice for 30 min, and cleared by centrifugation for 10 min at 10,000× g at 4 °C. Proteins were quantified by the Bio-Rad protein assay. Samples were subjected to SDS–PAGE as described in the previous section; 80 µg of protein were loaded per well.

### Cell proliferation by thymeleatoxin or siRNA-PKCα

RPE cells were cultured in 75 mm dishes (10×10^5^ cells/dish) in DMEM and allowed to grow to confluence. Then the cells were incubated with 100 nM thymeleatoxin for 24 h, or transfected with 100 nM siRNA using lipofectamine 2000 according to the manufacturer's protocol, with some modifications. The cells were fed with transfection reagent in serum-free DMEM for 24 h. Three independent siRNAs directed against PKC_α_ (A1–A3) were used, along with one control of scrambled siRNA (C): A1, dTd Ggc ugc uga cag aca ucu uu; A2, dCd Acc uac cau guu caa cga au; A3, dTd Acc gca gga caa cau acu uu; C, (product #2005527113152; Ruibo Biotech, Guangzhou, China). The medium containing thymeleatoxin or siRNA-PKC_α_was removed 24 h later.

### Immunofluorescence analysis

Human RPE cells grown on coverslips were stimulated for 24 h with 100 nM PMA, 100 nM thymeleatoxin, and 100 nM siRNA-PKC_α_. They were fixed for 15 min in PBS containing 4% paraformaldehyde, and then rinsed three times in PBS. All reagent incubations were performed in a humidified chamber. The primary antibodies were incubated for 16 h in a solution of PBS at room temperature; horse serum was used as the negative control instead of the primary antibody. After washing four times for 10 min in PBS, FITC and Cy3 -labeled secondary antibodies were incubated for 40 min at 37 °C. Then Hoechst 33342 was incubated for 5 min at room temperature. After three rinses in PBS, coverslips were mounted onto glass slides. Slides were analyzed on a Zeiss laser scanning confocal microscope (LSCM510META). Each antibody was used in a minimum of three separate experiments.

### Data and statistical analysis

Results are expressed as mean ±standard deviation (SD). Statistical analyses were performed upon comparisons using one-way ANOVA (ANOVA). A value of p<0.05 was considered significant.

## Results

### PKC activation with a phorbol ester affects cell cycle progression

**Figure 1:**
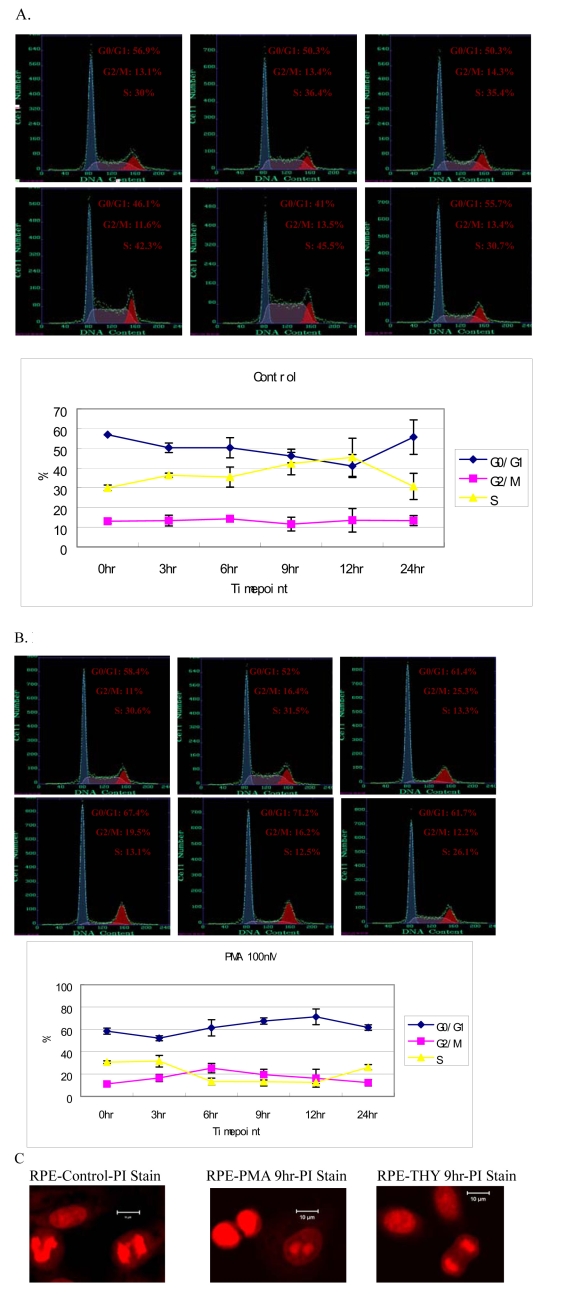
PKC activation with PMA affects cell cycle in human RPE cells. Flow cytometry analysis of PMA-treated RPE cells (**A**) shows decreased S phase and increased G_2_-M phases cell numbers when compared with that of untreated RPE cells (**B**). For each side scatter plot, the *y*-axis is the number of cells, while the *x*-axis is the DNA content. Values from each scatter plot are graphed below panels **A** and **B**. Similar results after PMA treatment were obtained in eight independent experiments. **C**: Immunofluorescence of cellular DNA stained with propidium iodide (PI) showed cells in interphase or at different stages of mitosis. RPE cells were grown on glass coverslips for 24 h, treated either with PMA or with thymeleatoxin for 9 h, and then fixed.

Previous work has shown that the PKC inhibitors, hypericin, and calphostin C, dramatically affect the growth rate of RPE cells [9,10]; however, the identity of the PKC isoform involved has remained unclear. To establish which isoform of PKC is potentially involved, we tested the effect of a phorbol ester, PMA, a potent activator of conventional and novel PKC isoforms, on the cell cycle progression of human RPE cells. As shown in Figure 1A,B, after 3 h following the addition of 100 nM PMA, RPE cells entered the S phase. The numbers that entered into S phase at 6, 9, and 12 h time points in PMA-treated RPE cells were decreased when compared with those in the control cells (p<0.05). In contrast, the numbers that entered into the G_2_-M phases of the cell cycle were increased between 3 and 12 h of treatment, indicating that PMA can slightly affect progression through the cell cycle. By 24 h of treatment, the distribution of the cells between the different phases of the cell cycle was similar to that of the control cells treated only with the vehicle dimethyl sulfoxide. However, control cells showed no significant change in distribution between the different phases of the cell cycle during the 24 h time course examined.

To further confirm that cells were not blocked in the G_2_ phase, PI-stained RPE cells grown on glass coverslips were analyzed by immunofluorescence microscopy at various times following PMA or vehicle treatment. Cells at all stages of mitosis could be observed in both PMA-treated cells and vehicle-treated cells, indicating that the cells were progressing normally through mitosis (Figure 1C). Therefore, PKC activation seemed to play a role in the regulation of cell cycle progression.

**Figure 2:**
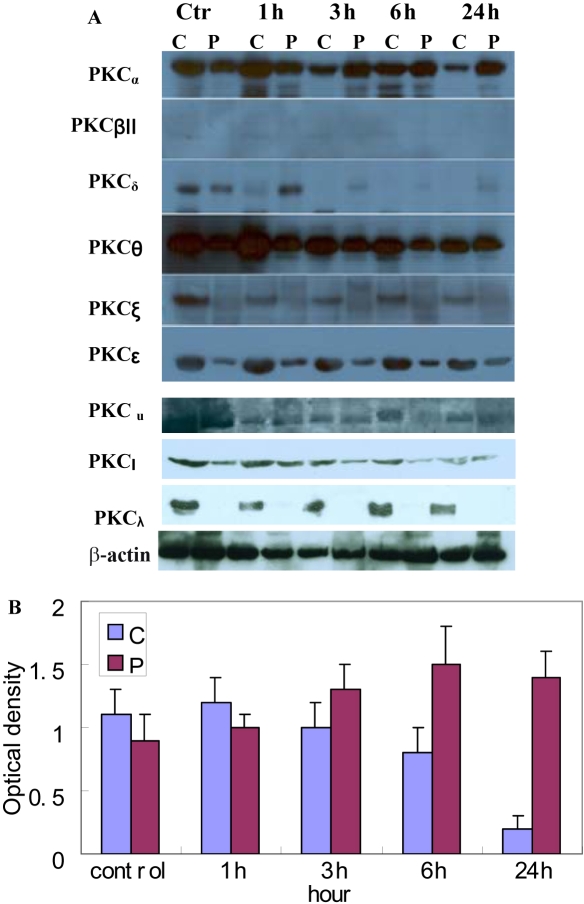
PKC_α_ and PKC_δ_, are the only isoforms translocated by PMA in RPE cells. **A**: Shown is a western blot analysis of the subcellular distribution between cytosolic and membrane fractions of the nine PKC isoforms expressed in RPE cells. RPE protein extracts were fractionated into cytosolic (C) and particulate (P) fractions; 80 µg of protein was loaded in each well. Only PKC_α_ and PKC_δ_ were translocated in response to PMA. PKC_δ_ was completely downregulated by proteolytic degradation by 6 h of treatment, while PKC_α_ was translocated from the cytosolic to the particulate fraction from 3 h to 24 h. Note that in the doublet obtained for PKC_δ_, only the upper band (78 kDa) is the active form of the enzyme. **B**: Optical density of PKC_α_ determined by densitometric imaging is shown (Mean±SD; n=4). The contents at different time points are statistically different (F=2.337, p<0.05). The β-actin band with 42 kDa is used for quantitation.

To identify the isoform of PKC responsible for the progression of the cells through the cell cycle, we monitored the effect of PMA on the subcellular localization of the nine PKC isoforms expressed in human RPE cells. Translocation of PKC from the cytosol to the membrane is a hallmark of its activation [25]. Upon PMA treatment, only PKC_α_ and PKC_δ_ were translocated from the cytosolic to the particulate fraction. PKC_βII_, PKC_ε_, PKC_θ_, PKC_ζ_, PKCι, PKC_λ_, and PKC_μ_ were not affected by PMA (Figure 2). PKC_δ_ was completely downregulated by proteolytic degradation by 6 h of treatment, while PKC_α_ was translocated from the cytosolic to the particulate fraction between 3 h to 24 h of treatment. Hence, the data indicate that of the nine PKC isoforms expressed in RPE cells, only PKC_α_ and PKC_δ_ were significantly activated by PMA stimulation.

### PKCα is necessary and sufficient to affect progression through the cell cycle

To differentiate between PKC_α_ and PKC_δ_, we used the conventional isoform-specific PKC agonist thymeleatoxin [26]. Since PKC_α_ is the only one of the three isoforms that translocates, it is likely that this agonist would only affect PKC_α_.

**Figure 3:**
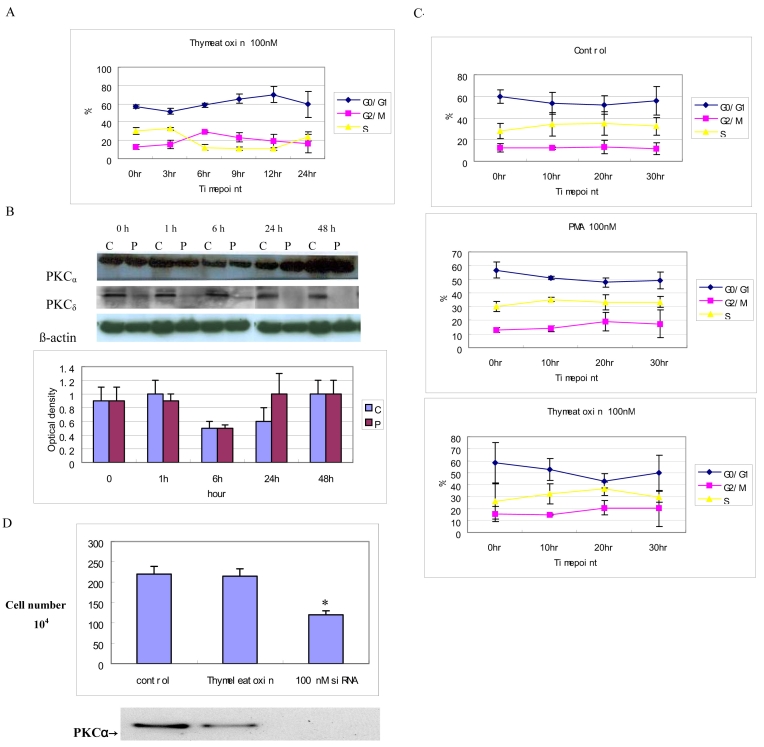
PKC_α_ is necessary and sufficient to affect cell cycle progression. **A**: Flow cytometry analysis of RPE cells after 100 nM thymeleatoxin treatment shows a cell cycle progression profile similar to that obtained with PMA in eight experiments. **B**: western blot analysis shows that PKC_α_ was rapidly translocated to the membrane by thymeleatoxin and downregulated within 24 h, the protein remained undetectable after 48 h of treatment, however, PKC_δ_ was not translocated and was not downregulated at all time points. Eighty micrograms of protein was loaded in each well. Optical density of PKC_α_ determined by densitometric imaging is shown (Mean±SD, n=3). The β-actin band with 42 kDa is used for quantitation. **C**: Flow cytometry analysis of RPE cells shows that there was no significant change in the cell cycle progression following PMA or thymeleatoxin restimulation when compared with the control over the 30 h time course after 48 h of PMA treatment. **D**: PKC_α_ activity regulates the growth rate of RPE cells. Approximately 110,000 RPE cells were seeded and then incubated with thymeleatoxin or siRNA-PKC_α_ for 24h. The numbers of cells were counted using a Coulter Counter and displayed in the top panel (* p<0.0001). Western blot using an anti-PKC_α_ antibody showed that the total PKC_α_ level was dramatically decreased in siRNA-PKC_α_ treated cells; 40 µg of protein was loaded in each well.

Flow cytometry analysis of 100 nM thymeleatoxin-treated RPE cells (Figure 3A) showed a cell cycle progression profile similar to that obtained with PMA (Figure 1A). Western blot analysis confirmed that PKC_α_ was specifically translocated (and activated) by thymeleatoxin, whereas PKC_δ_ remained unaffected (Figure 3B).

Further confirmation of the specific role of PKC_α_ in the regulation of the cell cycle progression of RPE cells was provided by PKC_α_ depletion experiments. RPE cells were pretreated with PMA for 48 h to deplete the cells of their endogenous PKC_α_. Cells were then restimulated with 100 nM PMA and 100 nM thymeleatoxin, and their distribution between the different phases of the cell cycle was analyzed between 0 and 30 h following restimulation by flow cytometry (Figure 3C). In the absence of a detectable level of PKC_α_, there was no significant change in the cell cycle progression of RPE cells following PMA or thymeleatoxin stimulation over the 30-h time course (Figure 3C), unlike the case of cells containing PKC_α_ (Figure 1).

Although PKC or PKC_α_ activation affects cell cycle progression, the proliferation of the PKC isoform involved has remained unclear. The role of PKC_α_ in cell proliferation was further addressed using siRNA or thymeleatoxin. Equally seeded cultures were grown and counted, giving a direct reading of their growth rate. SiRNA-PKC_α_ clones exhibited a growth rate of about half the rate of the control cells (Figure 3D), thus indicating that PKC_α_ levels are directly proportional to the basal proliferation rate of RPE cells. However, PKC_α_ agonist, thymeleatoxin, did not exhibit a growth rate of the RPE cells (Figure 3D), indicating that thymeleatoxin has no significant effect on cell proliferation. Altogether, the data strongly suggest that PKC_α_-specific activation is necessary and sufficient for the regulation of human RPE cells through the cell cycle. Moreover, our data indicate that PKC_α_ affects RPE cell proliferation, since decreased PKC_α_ expression correlates with decreased proliferation.

### p27^kip1^ mRNA and protein levels are downregulated following PKCα activation

**Figure 4:**
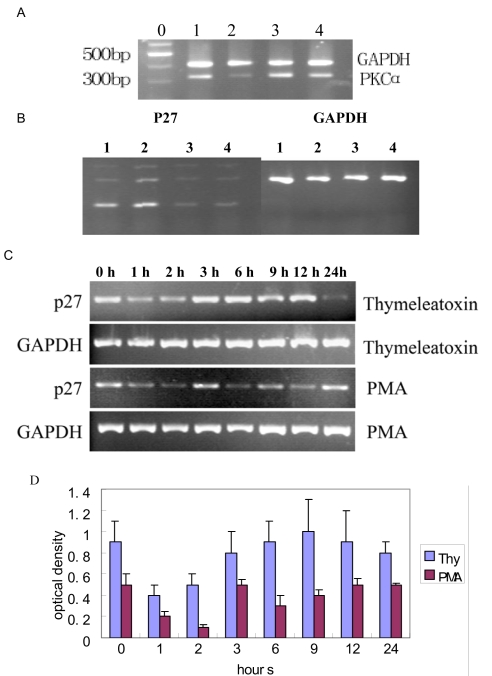
Downregulation of the p27^Kip1^ gene following PKC_α_ activation. **A**: PKC_α_ mRNA level is downregulated at the 3 h point following 24 h of siRNA-PKC_α_ (lane 2) treatment, but not upregulated following PMA (lane 4) and thymeleatoxi (lane 3) treatment. lane 1, control. **B**: p27^kip1^ mRNA level is downregulated following 3 h of PMA (lane 4) and thymeleatoxi (lane 3) treatment and upregulated following siRNA-PKC_α_ (lane 2) treatment. lane 1, control. Five micrograms of RNA was used for each reaction. **C**: RT–PCR analysis of RPE cells stimulated with 24 h PMA and thymeleatoxin treatment shows a strong downregulation of the p27 mRNA at 1, 2, and 6 h following PMA, or at 1 and 2 h following thymeleatoxin treatment. **D**: Optical density of P27 mRNA determined by densitometric imaging is shown (Mean±SD, n=4). The GAPDH band is used for quantitation.

As shown in Figure 4A, PKC_α_ can be downregulated at the 3-h time point following 24 h of siRNA-PKC_α_ treatment, but not upregulated following PMA and thymeleatoxi treatment. To evaluate the molecular mechanism by which PKC_α_ induces cell cycle progression, we used RT–PCR to examine the transcript levels of cell cycle-regulatory proteins after 3 h of treatment. We found that p27 mRNA was obviously downregulated following PMA or thymeleatoxin treatment, and upregulated following siRNA-PKC_α_ treatment. Levels of other mRNA (*CDK1, CDK2, CDK3, CDK4, Cyclin B1, Cyclin B2, Cyclin D2, Cyclin D3, Cyclin E1, Cyclin E2, p16, p18, p21*, and *Rb*) remained unaffected by PKC_α_ activation. The mRNAs for *CyclinA1, CyclinC, CyclinD1, p19*, and *p107*, could not be detected in this assay (Figure 4B). During 24 h of stimulation with PMA or thymeleatoxin, p27 mRNA was strongly and rapidly downregulated at 1, 2, and 6 h following PMA treatment, or at 1 h and 2 h following thymeleatoxin treatment (Figure 4C,D).

**Figure 5:**
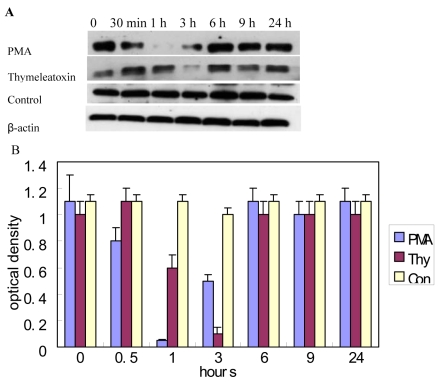
Downregulation of the p27^Kip1^ protein following PKC_α_ activation. **A**: Western blot analysis of RPE cells treated 24 h with 100 nM PMA and 100 nM thymeleatoxin reveals a strong downregulation of the p27 protein at the 1 h and 3 h time points. Gels show representative results of four independent experiments. Each well was loaded with 40 µg of protein. **B**: Optical density of P27 protein determined by densitometric imaging is shown (Mean±SD, n=4). The β-actin band with 42 kDa is used for quantitation.

Consistent with a change at the mRNA level, the p27 protein was also downregulated over a 24-h period following PMA or thymeleatoxin treatment of RPE cells (Figure 5). In untreated RPE cells, the p27 protein level remained constant, while in cells treated either with PMA or thymeleatoxin, p27 was strongly downregulated at the 1 h and 3 h time points. These data indicate that p27^kip1^ is the only cell cycle-regulatory molecule downregulated following PKC_α_ activation.

### Immunofluorescence colocalization of PKC_α_ and P27

**Figure 6:**
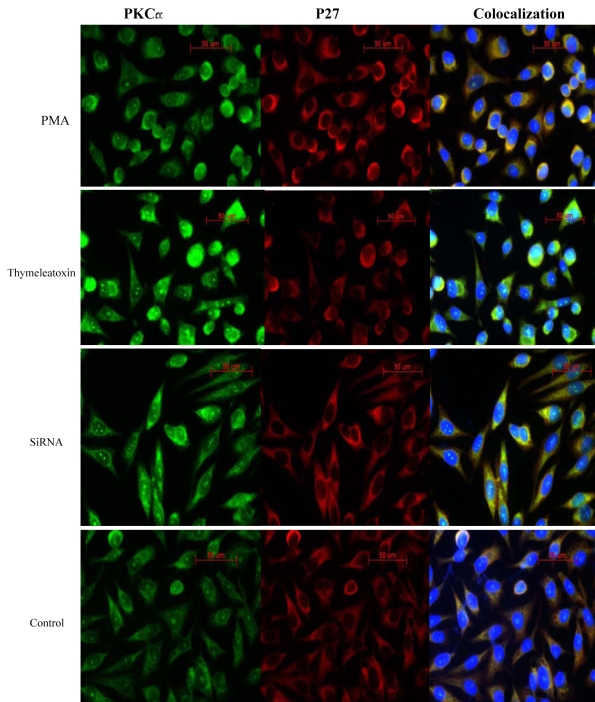
Confocal images of p27 and PKC_α_ colocaliztion in RPE cells. PKC_α_ and p27 have obvious cytoplasmic localizations and slight nuclear localization, and mostly colocalized in the cytoplasm of the cells stimulated with PMA, thymeleatoxin, and siRNA- PKC_α_. PKC_α_ (FITC, green label) p27 (Cy3, red label), nuclei (Hotchest 33342, blue label), PKC_α_, and p27 colocalization (yellow label).

Confocal microscopy clearly showed that the cultured RPE cells formed monolayers with typical polygonal cellular arrays. As shown in Figure 6, PKC_α_ and p27 have obvious cytoplasmic localizations and slight nuclear localization, and mostly colocalized in the cytoplasm of the cells. Although minor staining differences appeared among 100 nM PMA, 100 nM thymeleatoxin, and 100 nM siRNA-PKC_α_ when compared with that of control group, PKC_α_ and p27 seemed colocalized in the cytoplasm of the cells with decreased cell numbers in siRNA-PKC_α_.

## Discussion

### Effect of PKCα on the cell cycle progression in RPE cells

We have found that PKC activation by phorbol esters affected RPE cell progression through the cell cycle. This was consistent with previous data showing the correlation between the results on PKC activity and RPE cell proliferation [10] and the animal results that demonstrated inhibitors of PKC hypericin could have efficacy in rabbits with PVR [16,17]. Moreover, we have determined that only PKC_α_ activation is necessary and sufficient to regulate cell cycle progression of RPE cells, and that the expression level of PKC_α_ correlates with the proliferation of RPE cells.

It is well known that PKC has been associated with the regulation of cell cycle progression either during the G_1_-to-S progression or during the G_2_/M transition [27-29]. PKC has been shown to regulate G_1_ progression through the modulation of CDK activity, either by modifying cyclin or CDK expression levels, or by modifying the expression of the cyclin-CDK inhibitors. Due to the relevance of PKC isozymes in the control of cell cycles, both in G_1_/S and in G_2_/M, the elucidation of such complex intracellular networks using cellular and animal models has become of the outmost importance.

Predominantly, PKC plays an inhibitory role in many cell cycle progressions [23,30,31]. In intestinal epithelial cells, for instance, PKC_α_-specific activation resulted in G_1_ arrest and delayed transit through the S and G_2_/M phases through an upregulation of p21 and p27, resulting in hypophosphorylation of Rb [23]. However, in contrast to most cell types, phorbol esters accelerated growth factor-induced Swiss 3T3 cell cycle entry and progression into the S phase by elevating cyclin D1 levels and downregulating p27^Kip1^ expression [32]. In human RPE cells, ethambutol may exert toxic effects in RPE, including the suppression of cell growth, formation of cytoplasmic vacuoles, and reduction of phagocytic functions via the PKC signal pathway [33]. Alkylphosphocholines inhibit proliferation of RPE cells and RPE-mediated matrix contraction in vitro at nontoxic concentrations through the inhibition of PKC activity [34].

Our study is the first to show that the activation of PKC, and specifically PKC_α_, exerts effects on the S to G_2_/M progression of the human RPE cell cycle, as shown in Figure 1A and Figure 3A, and that the inhibition of PKC decreases the proliferation, as shown in Figure 3D. Recently, aprinocarsen, an antisense oligonucleotide (ASO) against PKC_α_, has been used to decrease the malignant proliferation in clinic trials in different cancers [35-39]. Similarly, since PKC_α_ was the only isoform associated with the proliferation of RPE cells in our study, it may be a rational approach for targeted therapies against RPE cell proliferation and PVR disease.

### Downpregulation of p27^kip1^ following PKC_α_ activation in RPE cells

To elucidate the mechanism by which PKC increased the RPE cell cycle progression, we analyzed the expression of various cell cycle-regulatory proteins following PKC activation. We found that the only cell cycle regulatory protein downregulated by PKC_α_ activity was the inhibitor p27^kip1^, which has been proposed to be part of a cell-intrinsic timer that arrests the cell cycle and initiates differentiation in several lineages [40,41].

Several groups have reported gigantism and multiple organ hyperplasia in mice with targeted disruption of the p27^kip1^ gene [42-47]. Some of the most dramatic phenotypic changes in these animals were involved in the retina. For example, Nakayama et al. reported that the RPE exhibited an increase in thickness in its apical to basal dimension compared to that seen in the congenic C57BL/6J strain [44]. Other results showed that the neural retina exhibited focal areas of dysplasia, attributed to extended histogenesis of photoreceptors and Müller cells and to the displacement of reactive glia into the layer of photoreceptor outer segments, leading to a disruption in the normal organization of the outer nuclear layer [45,46]. Defoe et al. examined the retinas of p27^kip1^ knockout mice in more detail and not only found that p27^kip1^ was an important factor in regulating RPE proliferation during development, but also observed that this protein may be a crucial factor involved in generating appropriately polarized epithelial cells and in the construction of the photoreceptor-RPE interface [47]. From these results, it was concluded that p27^kip1^ downregulation may be involved in the process of RPE cell proliferation and PVR disease. In our study, p27^kip1^ was downregulated by PKC_α_ activation at the gene and protein levels in RPE cells. Moreover, p27^kip1^ and PKC_α_ colocalized within the cells, as shown in Figure 6. Therefore, inhibitors of PKC_α_ could have antiproliferative effects on RPE cells in vitro and as a potential therapeutic drug for PVR via p27^kip1^ downregulation. In addition, a possible role of PKC_δ_ in cell cycle progression and proliferation in RPE cells should be ruled out in the future study.

**Figure 7:**
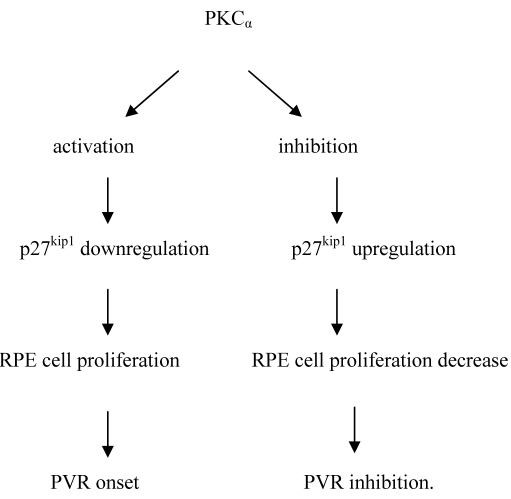
Proposed mechanisms of PKC_α_ regulation on PVR. PKC_α_ affects cell proliferation and PVR in human RPE cells through negative feedback of p27^kip1^.

On the other hand, several studies have reported the PKC-induced upregulation of p27 in other cell types [48-50]; however, this was associated with cell cycle blocks, unlike the case for RPE cells reported here. Taken together, the relationship between PKC_α_, p27^kip1^, and PVR is illustrated in Figure 7.

It is well known that PVR is a result of various biologic reactions, such as the synthesis of the extracellular matrix, contraction of membranes, and apoptotic change of photoreceptors. Of all the cells involved in PVR, the RPE cell is a central player, but the inhibition of RPE cell proliferation is not sufficient to inhibit PVR, which has been proven by studies of anticancer drugs for PVR since the 1990s.

In summary, we have found that PKC_α_ affects the cell cycle progression and proliferation in RPE cells through the downregulation of p27^kip1^. These results suggest that PKC_α_ can be used as a potential therapeutic target against RPE cell proliferation and PVR disease.
